# *Pharmaceutics* 2022 Best Paper Awards

**DOI:** 10.3390/pharmaceutics14091839

**Published:** 2022-08-31

**Authors:** Pharmaceutics Editorial Office

**Affiliations:** MDPI AG, St. Alban-Anlage 66, 4052 Basel, Switzerland; pharmaceutics@mdpi.com

*Pharmaceutics* [1] is instituting the Best Paper Awards to recognize outstanding papers published in the journal. We are now pleased to announce the winners of the “Pharmaceutics 2022 Best Paper Awards”.

The papers published in 2020 were preselected by the *Pharmaceutics* Editorial Office based on the number of citations and downloads from the website. The winners from the nominations were determined by an award committee, the Editor-in-Chief, and the Editorial Office. The following three top-voted papers, in no particular order, have won the *Pharmaceutics* 2022 Best Paper Awards:

## 1. Research Article

**Development of Remdesivir as a Dry Powder for Inhalation by Thin Film Freezing**

**Figure 1:**
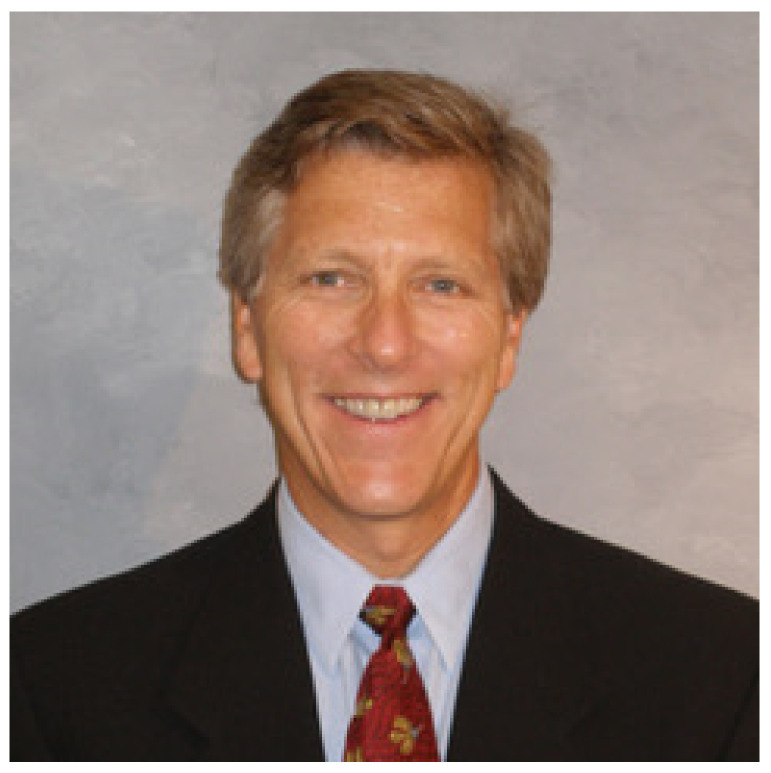
Robert O. Williams III.

By Sahakijpijarn, S.; Moon, C.; Koleng, J.J.; Christensen, D.J.; Williams, R.O., III. (Figure 1).

*Pharmaceutics* 2020, 12, 1002. https://doi.org/10.3390/pharmaceutics12111002.

Available online: https://www.mdpi.com/1999-4923/12/11/1002 (accessed on 27 July 2022).

Synopsis of the paper by the authors:

Remdesivir exhibits in vitro activity against SARS-CoV-2 and was granted approval for emergency use. To maximize its delivery to the lungs, we formulated remdesivir as a dry powder for inhalation using thin film freezing (TFF). TFF produces brittle matrix-nanostructured aggregates that are sheared into respirable low-density microparticles upon aerosolization from a passive dry powder inhaler. In vitro aerodynamic testing demonstrated that the drug loading and excipient type affected the aerosol performance of remdesivir. Remdesivir combined with optimal excipients exhibited a desirable aerosol performance (up to 93.0% FPF < 5 µm; 0.82 µm mass median aerodynamic diameter). Remdesivir was amorphous after the TFF process, which benefitted the drug’s dissolution in simulated lung fluid. TFF remdesivir formulations are stable after one month of storage at 25 °C/60% relative humidity. An in vivo pharmacokinetic evaluation showed that TFF remdesivir–leucine was poorly absorbed into systemic circulation while TFF remdesivir-Captisol^®^ demonstrated an increased systemic uptake compared to leucine. Remdesivir was hydrolyzed to the nucleoside analog GS-441524 in the lung, and the levels of GS-441524 were greater in the lung with leucine formulation compared to Captisol^®^. In conclusion, TFF technology produces high-potency remdesivir dry powder formulations for inhalation that are suitable to treat patients with COVID-19 on an outpatient basis and earlier in the disease course when effective antiviral therapy can reduce the related morbidity and mortality [2].

## 2. Research Article

**Manufacturing Considerations for the Development of Lipid Nanoparticles Using Microfluidics**

**Figure 2:**
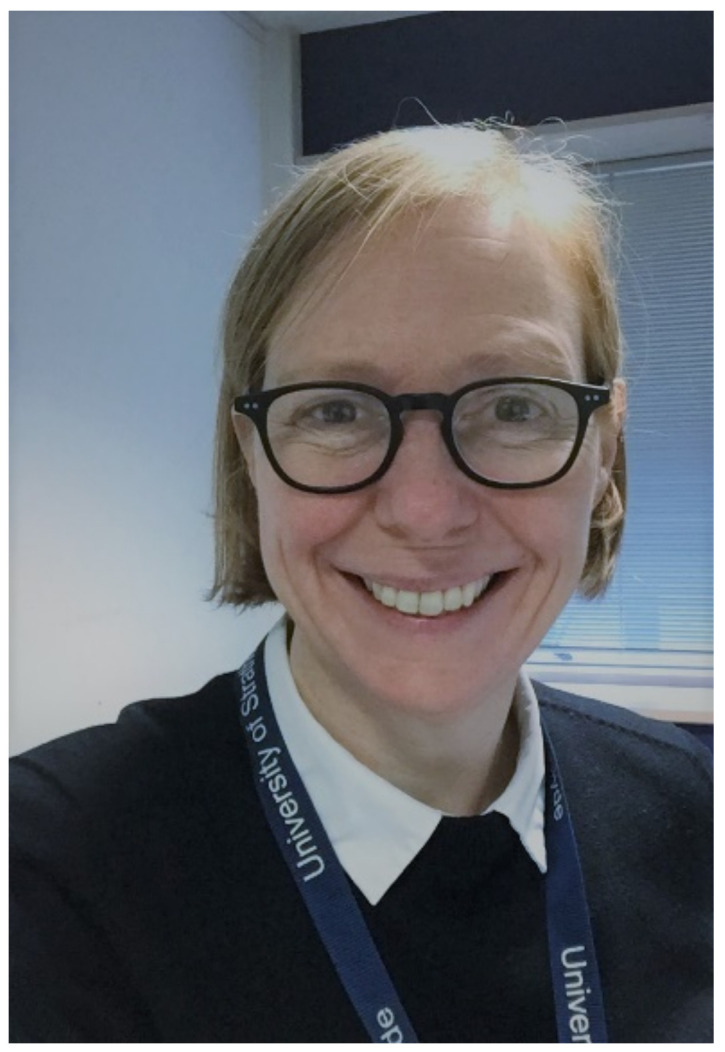
Yvonne Perrie.

By Roces, C.B.; Lou, G.; Jain, N.; Abraham, S.; Thomas, A.; Halbert, G.W.; Perrie, Y. (Figure 2).

*Pharmaceutics* 2020, 12, 1095. https://doi.org/10.3390/pharmaceutics12111095.

Available online: https://www.mdpi.com/1999-4923/12/11/1095 (accessed on 27 July 2022).

Synopsis of the paper by the authors:

In recent years, the use of lipid nanoparticles (LNPs) for RNA delivery has gained considerable attention, with a large number in the clinical pipeline as vaccine candidates or for treating a wide range of diseases. Microfluidics offer considerable advantages for their manufacture due to their scalability, reproducibility, and fast preparation. Thus, in this study, we have evaluated the operational and formulation parameters to be considered when developing LNPs. Among them, the flow rate ratio (FRR) and the total flow rate (TFR) have been shown to significantly influence the physicochemical characteristics of the produced particles. In particular, increasing the TFR or increasing the FRR decreased the particle size. The amino lipid choice (cationic—DOTAP and DDAB; ionisable—MC3), buffer choice (citrate buffer pH 6 or TRIS pH 7.4), and type of nucleic acid payload (PolyA, ssDNA or mRNA) have also been shown to have an impact on the characteristics of these LNPs. LNPs were shown to have a high (>90%) loading in all cases and were below 100 nm with a low polydispersity index (≤0.25). The results within this paper could be used as a guide for the development and scalable manufacture of LNP systems using microfluidics [3].

## 3. Review

**Skin Wound Healing Process and New Emerging Technologies for Skin Wound Care and Regeneration**

**Figure 3:**
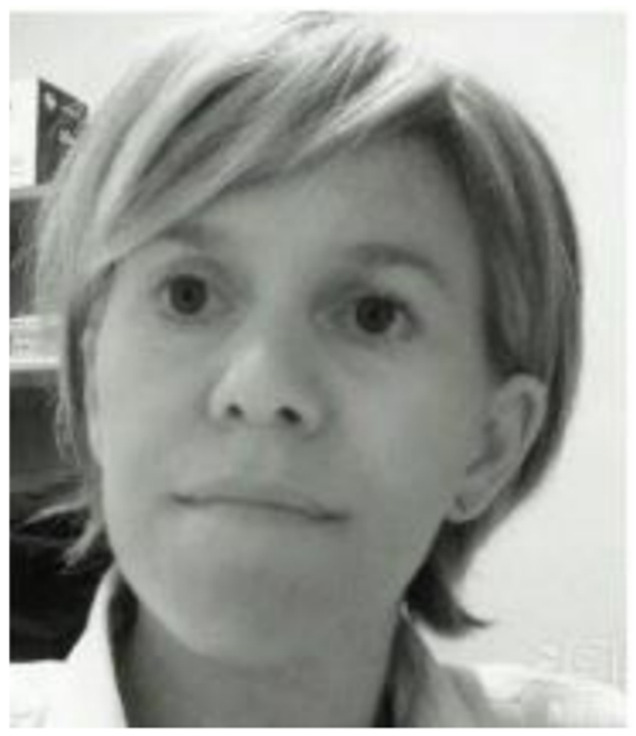
Rossella Dorati.

By Tottoli, E.M.; Dorati, R.; Genta, I.; Chiesa, E.; Pisani, S.; Conti, B. (Figure 3).

*Pharmaceutics* 2020, 12, 735. https://doi.org/10.3390/pharmaceutics12080735.

Available online: https://www.mdpi.com/1999-4923/12/8/735 (accessed on 27 July 2022).

Synopsis of the paper by the authors:

Skin wound healing shows an extraordinary cellular function mechanism, which is unique in nature and involves the interaction of several cells, growth factors, and cytokines. Physiological wound healing restores tissue integrity, but in many cases the process is limited to wound repair. Ongoing studies aim to obtain more effective wound therapies with the intention of reducing inpatient costs, providing long-term relief, and enabling effective scar healing. The main goal of this comprehensive review is to focus on the progress in wound medication and how it has evolved over the years. The main complications related to the healing process and the clinical management of chronic wounds are described in the review. Moreover, the advanced treatment strategies for skin regeneration and experimental techniques for cellular engineering and skin tissue engineering are addressed. Emerging skin regeneration techniques involving scaffolds activated with growth factors, bioactive molecules, and genetically modified cells are exploited to overcome wound-healing technology’s limitations and to implement personalized therapy designs [4].

These three papers represent valuable contributions to *Pharmaceutics*. We warmly congratulate both teams on their accomplishments and wish them continued success.

*Pharmaceutics* 2022 Best Paper Awards Committee,

*Pharmaceutics* Editorial Board.
